# Déficit congénital en facteur VII de coagulation: à propos de deux cas familiaux

**DOI:** 10.11604/pamj.2018.31.156.6123

**Published:** 2018-10-31

**Authors:** Noufissa Benajiba, Anass Ayyad, Chourouk Aabdi, Rim Amrani, Maria Rkain, Mohammed Benajiba

**Affiliations:** 1 Service de Pédiatrie, Hôpital Al Farabi, CHU Mohamed VI, Faculté de Médecine et de Pharmacie, Université Mohammed Premier, Oujda, Maroc; 2 Centre National de Transfusion Sanguine, Rabat, Maroc

**Keywords:** Déficit congénital, Facteur VII, mariage non consanguin, Congenital, factor VII deficiency, non-consanguineous marriage

## Abstract

Le déficit en facteur VII est rare, sa prévalence est estimée à 1/1.000.000. Sa transmission est autosomique récessive. L'expression peut aller de la simple épistaxis à l'hémorragie cérébrale. Le but de notre travail est de mettre l'accent sur les particularités cliniques et l'intérêt du dépistage de ce rare déficit. Nous rapportons deux observations de deux frères porteurs de ce déficit. Il s'agit d'un enfant âgé de 8 ans, issu d'un mariage non consanguin, benjamin d'une fratrie de deux, dans ses antécédents on note une hémorragie post circoncisionnelle, admis au service pour prise en charge d'épistaxis à répétition depuis l'âge de 4ans. Un bilan d'hémostase a été réalisé objectivant un taux de prothrombine (TP) bas, un temps de céphaline activée (TCA) normal et le dosage factoriel a révélé un déficit en facteur VII avec un taux à 26%. L'évolution est marquée par des transfusions espacées du plasma frais congelé (PFC) suite à des épistaxis et des plaies. Le dépistage familial n'a pas été réalisé. Son frère ainé, consulte à l'âge de 11 ans pour épistaxis de grande abondance, l'examen somatique a été sans particularité. Vu ses antécédents, le patient a bénéficié d'un bilan avec dosage du facteur VII révélant un taux à: 55%, et un dépistage chez les parents est prévu. La découverte d'un cas doit motiver à mener une enquête familiale afin de dépister d'autres porteurs de ce déficit et avoir un conseil génétique qui aidera à éviter des manifestations graves voir mortelles tout en sachant que les études n'ont pas montré de corrélation entre le taux du facteur et la gravité du tableau.

## Abstract (en)

Factor VII deficiency is rare, with an estimated prevalence rate of 1/1,000,000. It is transmitted as an autosomal recessive trait. It can cause simple nosebleeds up to cerebral hemorrhage. Our study aims to focus on the clinical features and the importance of screening in patients with this rare deficit. We report the cases of two brothers with this deficit. Child aged 8 years, born to non-consanguineous marriage who was the youngest of two children. He had a history of post-circumcision bleeding and was admitted to our Department for the treatment of recurrent nosebleeds occurred over the last 4 years. Screening tests of hemostasis showed low Prothrombin (PT), normal Activated thromboplastin time (ATT), while factor assay revealed factor VII deficiency with a rate of 26%. The patient underwent spaced fresh frozen plasma (FFP) transfusions due to nosebleeds and wounds. Family screening was not performed. The eldest brother, aged 11 years, presented with very abundant nosebleeds. Somatic examination was unremarkable. Given his history, the patient underwent factor VII assay revealing a rate of 55% and parent screening was scheduled. The diagnosis of congenital factor VII deficiency in a patient motivates family screening in order to perform screening tests in other carriers of factor VII deficiency. This would avoid severe manifestations, even fatal, considering that studies have not shown a correlation between factor VII rate and the severity of patient’s status.

## Introduction

Le facteur VII de la coagulation ou proconvertine est une glycoprotéine du sang, synthétisée par le foie. Il intervient dans la voie exogène de la coagulation. Le déficit congénital en facteur VII est rare, sa prévalence est estimée à 1/1.000.000. Sa transmission est autosomique récessive. L'expression peut aller de la simple épistaxis à l'hémorragie cérébrale [1, 2]. Le traitement consiste à l'administration de concentré de facteur VII. L'objectif de notre travail et de mettre l'accent sur les particularités cliniques de ce rare déficit, et l'intérêt du dépistage autour d'un cas.

## Patient et observation

**Observation 1:** il s'agit d'un garçon âgé de 8 ans, issu d'un mariage non consanguin; benjamin d'une fratrie de deux. Il a été référé par un ORL au service de pédiatrie pour prise en charge d'épistaxis récidivantes depuis l'âge de 4 ans. L'anamnèse a objectivé la notion d'une hémorragie postcirconcisionnelle non bilantée à l'âge de 2 ans, par ailleurs il n'y a pas de cas similaire rapporté dans la famille. L'examen clinique était normal. Devant le syndrome hémorragique fait d'hémorragie de circoncision et d'épistaxis récidivants, une cause générale a été suspectée notamment un trouble d'hémostase. Un bilan d'hémostase a été réalisé objectivant un TCA normal, un TP bas et le dosage factoriel a révélé un déficit en facteur VII avec un taux à 26% (valeurs normales: 70- 140%). Le déficit en facteur VII a été retenu. Le traitement instauré chez notre patient était la transfusion de plasma frais congelé, vu le cout élevé du facteur VII recombinant activé. Le dépistage familial n'a pas été réalisé.

**Observation 2:** le frère ainé âgé de 11 ans a présenté des épistaxis de grande abondance motivant la consultation dans notre formation où un examen somatique complet a été réalisé revenant normal. Vu les antécédents familiaux, le patient a bénéficié d'un bilan d'hémostase avec dosage du facteur VII révélant un taux à: 55%. Le déficit en facteur VII a été également retenu et le patient a reçu également du plasma frais congelé.

## Discussion

Le déficit en facteur VII a été décrit pour la première fois en 1951 par Alexander. C'est un déficit rare, dont la prévalence est estimée à 1/500.000 à 1/1.000.000 [1, 3], en revanche une personne sur 500 peut être porteuse du gène [4]. Le déficit en facteur VII est un trouble autosomique récessif, cela signifie également qu'il atteint tant les filles que les garçons. Le gène responsable est localisé sur le chromosome 13 [5]. Le déficit en facteur VII est très rare, mais comme tous les troubles autosomiques récessifs, il est plus répandu là où les mariages consanguins sont fréquents d'où l'intérêt d'établir une stratégie de dépistage pour un diagnostic précoce. Le déficit en facteur VII forme un groupe hétérogène sur le plan génotypique et phénotypique [5]. L'âge de révélation est variable: plus il est précoce plus l'hémorragie est spontanée et grave. La symptomatologie clinique est aussi variée allant d'une simple épistaxis jusqu'à l'hémorragie cérébrale: le déficit peut se révéler à l'âge néonatal par une hémorragie à la chute du cordon, dans l'enfance par un saignement lors de la chute des dents de lait, à la puberté par des ménorragies chez une fille [2]. Le déficit peut rester inaperçu et n'apparaitre qu'à l'âge adulte suite à un traumatisme ou à un acte chirurgical, comme il peut se révéler par des hémarthroses [6]. Par ailleurs, des cas d'accidents thrombotiques ont été rapportés dans la littérature chez des porteurs du déficit [5]. Dans les deux cas que nous rapportons le déficit congénital en facteur VII s'est révélé dès la petite enfance chez le benjamin par une hémorragie post circoncisionnelle et des épistaxis alors que chez l'ainé il ne s'est révélé qu'à la grande enfance par un saignement muqueux. La corrélation entre la profondeur du déficit et la sévérité du saignement n'est pas prouvée [1, 5], malgré que certains auteurs rapportent que plus le facteur est bas plus les saignements sont graves [2]. D'où l'intérêt d'un dépistage autour d'un cas. Le déficit en facteur VII est suspecté devant la combinaison d'un temps de Quick allongé et d'un TCA normal [5, 7]. Le dosage du facteur VII constitue le moyen de ce dépistage. Les valeurs normales sont comprises entre 70% et 140%. La forme homozygote se définit par un taux de proconvertine anormalement bas inférieur à 10%, alors que la forme hétérozygote se définit par des taux à la limite inférieure du taux normal du facteur VII [2, 5]. Tel le cas de nos patients qui sont probablement hétérozygote. Le dépistage chez les parents a été proposé. L'intérêt du dépistage familial permet de diagnostiquer aussi bien les formes homozygotes que les formes hétérozygotes qui peuvent rester asymptomatiques dans la majorité des cas, comme ils peuvent manifester un syndrome hémorragique, tel le cas de notre patient, ainsi, ce dépistage permet d'établir une stratégie de prévention et de surveillance. Le traitement n'est indiqué qu'en cas d'accidents hémorragiques. Plusieurs alternatives thérapeutiques sont disponibles, dont le facteur VII recombinant activé qui possède une excellente efficacité et une très bonne tolérance, la dose usuelle recommandée en cas de saignement est de 15 à 30 ug/kg toutes les 4 à 6h jusqu'à arrêt de l'hémorragie. En préopératoire la prophylaxie est indiquée en raison de 20 à 30 ug/Kg de facteur VII en préopératoire et 5 à 10 ug/Kg toutes les 4 à 6h en post opératoire pendant 5 à 10 j [2, 5, 8]. Un traitement substitutif au long cours a été proposé par certains auteurs en cas de saignements graves et répétés à raison de deux injections par semaine [2, 8]. A défaut du facteur VII, vu son cout élevé, nous continuons à administrer du plasma frais congelé en cas d'hémorragie tel le cas pour nos patients qui consultent au moindre saignement et chez qui le seul traitement disponible est la transfusion du plasma qui reste d'un grand apport dans notre contexte, tout en sachant que son efficacité est faible, en plus du risque de transmission infectieuse.

## Conclusion

Le déficit en facteur VII est une affection héréditaire rare pouvant mettre en jeu le pronostic vital. La découverte d'un cas impose le dépistage familial pour une meilleure prévention et surveillance.

## Conflits d’intérêts

Les auteurs ne déclarent aucun conflit d’intérêts.
